# Bibliometric and visualization analysis of research trend in mental health problems of children and adolescents during the COVID-19 pandemic

**DOI:** 10.3389/fpubh.2022.1040676

**Published:** 2023-01-06

**Authors:** Zeming Guo, Yiran Zhang, Qin Liu

**Affiliations:** School of Public Health, Chongqing Medical University, Chongqing, China

**Keywords:** COVID-19, children, adolescent, mental health, Citespace, bibliometric analysis

## Abstract

**Objectives:**

To analyze the evolution of research on children and adolescents mental health issues during COVID-19 pandemic and discuss research hotspots and cutting-edge developments.

**Methods:**

The literature obtained from the web of science core collection as of June 28, 2022, was analyzed using Citespace, VOSviewer bibliometric visualization mapping software.

**Results:**

A total of 6,039 relevant papers were found, of which 5,594 were included in the study. The number of literatures is growing since 2020; and the country, institution, and journal publications were analyzed. The co-citation analysis shows that there are more research articles among the highly cited articles and a lack of systematic reviews that use critical thinking for review. In the cluster analysis, mental health and life change were the most representative. The timeline view of the keywords shows that Online learning (#0), Public health (#1), and Mental health (#2) are the three largest clusters and shows the change over time.

**Conclusion:**

This study helped analyze the mental health of children and adolescents during the COVID-19 pandemic and identified hot trends and shortcomings, which are important references for the theoretical basis of future research and decision making and technical guidance for systematic reviews.

## 1. Introduction

Since the first case of unexplained pneumonia was reported and identified as 2019-nCoV in Wuhan, Hubei Province, China, in December 2019, the novel coronavirus pneumonia outbreak has assumed a global pandemic pattern (1). The emergence of COVID-19 has resulted in the most serious global infectious disease pandemic in nearly 100 years, resulting in a huge development challenge for all of humanity. COVID-19 causes human-to-human transmission primarily through respiratory infections, and countries have taken extensive measures to reduce the spread of COVID-19 in order to control the epidemic. At the meantime, countries worldwide are concerned about the potential impact of the COVID-19 epidemic in other aspects.

To control the further spread of the infection, many governments have issued disease prevention and control measures, mainly through quarantine of patients, asymptomatic infected persons and close contacts, following by targeted treatment of patients according to the features of COVID-19 (2). Further transmission of COVID-19 is effectively controlled through quarantine measures, but the mental health effects on people in an outbreak quarantine situation should also be taken into consideration. There will be negative psychological effects on people experiencing quarantine, including post-traumatic stress symptoms, confusion, and anger. Stressors include longer periods of quarantine, fear of infection, depression, boredom, inadequate supplies, lack of information, financial loss, and stigma (3). A more important but easily overlooked issue is the mental health impact on children and adolescents under the COVID-19 pandemic. Children and adolescents are more likely to be affected by environmental factors than adult. Sprang and Silman have shown posttraumatic stress symptoms in isolated children by comparing parents and isolated children with non-quarantined children, suggesting that the average posttraumatic stress score of isolated children was four times higher than that of non-isolated children (4). Children and adolescents in prolonged home quarantine may experience increased loneliness and therefore increased mental health problems in children and adolescents due to social restrictions and school closures (5). In addition, the interaction between psychosocial stress caused by lifestyle changes in the context of the pandemic may further exacerbate the adverse effects on children's physical and mental health, leading to a vicious cycle (6). Children and adolescents will have a higher risk of mental health problems under the pandemic, so it is of research significance to focus on mental health problems of children and adolescents and propose effective solutions.

The purpose of this paper is to conduct a bibliometric and visualization analysis of the distribution and trends of research on mental health issues in children and adolescents during the COVID-19 pandemic. This study will help researchers to better understand the current research progress and identify future research directions. This paper explores the following questions: (1) What are the general publication trends worldwide for research on mental health issues in children and adolescents in the context of the COVID-19 pandemic? (2) Which countries or regions have been dominant in this research direction? (3) Which journals, institutions, and authors are most influential in this area of research? (4) What are the current research hotspots on the mental health of children and adolescents during the COVID-19 pandemic? (5) What are the future trends in mental health of children and adolescents? (6) What recommendations can be made to scholars and policy makers?

## 2. Methods

### 2.1. Data source

This study searched the web of science core collection, a database commonly used in bibliometric analysis, which contains the most important and commonly used journals in the world, covering a wide range of fields, with the neutrality and informativeness needed for analysis (7–9).

### 2.2. Methodology

**Figure 1:**
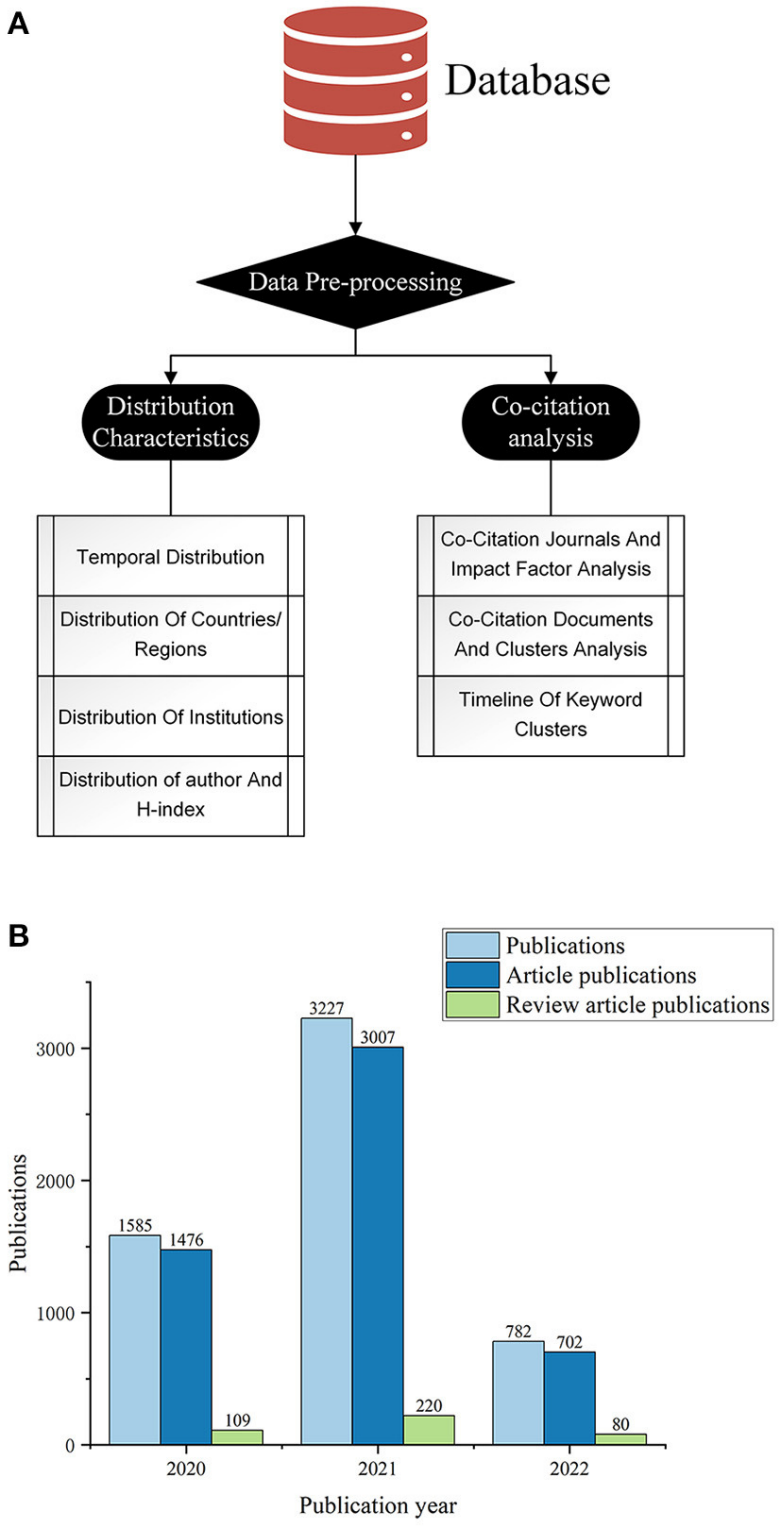
Bibliometric and visualization analysis framework **(A)** and Annual publication distribution **(B)**.

Scientific mapping is an important method in bibliometric analysis (10) and can help to analyze and visualize a field in order to obtain revolutionary changes and new trends in the structure of the field (11). In this paper, we used CiteSpace (Chaomei Chen, China) (12, 13), VOSviewer (14) (Centre for Science and Technology Studies, Leiden University, The Netherlands), Scimago Graphica (Scimago Lab, Portugal) to create visualization maps. Co-citation networks analysis and co-occurrence keyword analysis were performed using time slices from Citespace. Origin 2022 (Origin Lab, Northampton, Massachusetts, USA.) was used to plot histograms. The analysis process is shown in Figure 1A.

Using bibliometric methods and knowledge mapping visualization software, we analyzed the distribution and development trends of studies on mental health issues in children and adolescents during COVID-19 pandemic. Our search terms were set as followed. TS = (“coronavirus 2019” OR “COVID 19” OR “coronavirus disease 2019” OR “2019 novel coronavirus” OR “2019-novel CoV” OR “COVID 2019” OR “2019 ncov” OR “COVID19” OR “nCoV-2019” OR “nCoV2019” OR “nCoV 2019” OR “COVID-19” OR “Severe acute respiratory syndrome coronavirus 2” OR “2019-ncov” OR “SARS-CoV-2”) AND TS = (“children” OR adolescen^*^ OR “student”) AND TS = (“mental” OR “psychological” OR “psychiatry” or “psychiatric” OR “emotional” OR “stress” “stressed” OR “stressful” OR “anxiety” OR “anxious” OR “depression” OR “depressed” OR “depressive” OR “depress” OR “anger” OR “angry” OR “loneliness” OR “lonely” OR “burnout” OR “insomnia” OR “fear” OR “worry” OR “frustration” OR “posttraumatic stress disorder” OR “post-traumatic stress” OR “posttraumatic stress” OR “PTSD”).

The inclusion criteria used to determine the studies in this study were (1) studies about mental health in children and adolescent during COVID-19 pandemic, (2) research articles and review articles (3) published in English in all years. All literature was downloaded on June 28, 2022, with a total of 6039 literature obtained. After screening, a total of 5594 studies from the perspective of mental health of children and adolescent were included.

## 3. Distribution characteristics

### 3.1. Temporal distribution

Among the 5,594 studies included, 409 were review articles and 5,185 were research articles. Figure 1B shows the distribution of publications in the 3 years since the outbreak of COVID-19. The first literature was published by Rehma (15) in 2020, suggesting the need for mental health support for children in Pakistan during the COVID-19 pandemic. Since the onset of the new crown pneumonia outbreak in 2020, there has been a growing interest in mental health issues for children and adolescents, however, it has been a relatively short period of time and is still in its infancy.

### 3.2. Global geographic distribution

#### 3.2.1. Distribution of countries/regions

**Figure 2:**
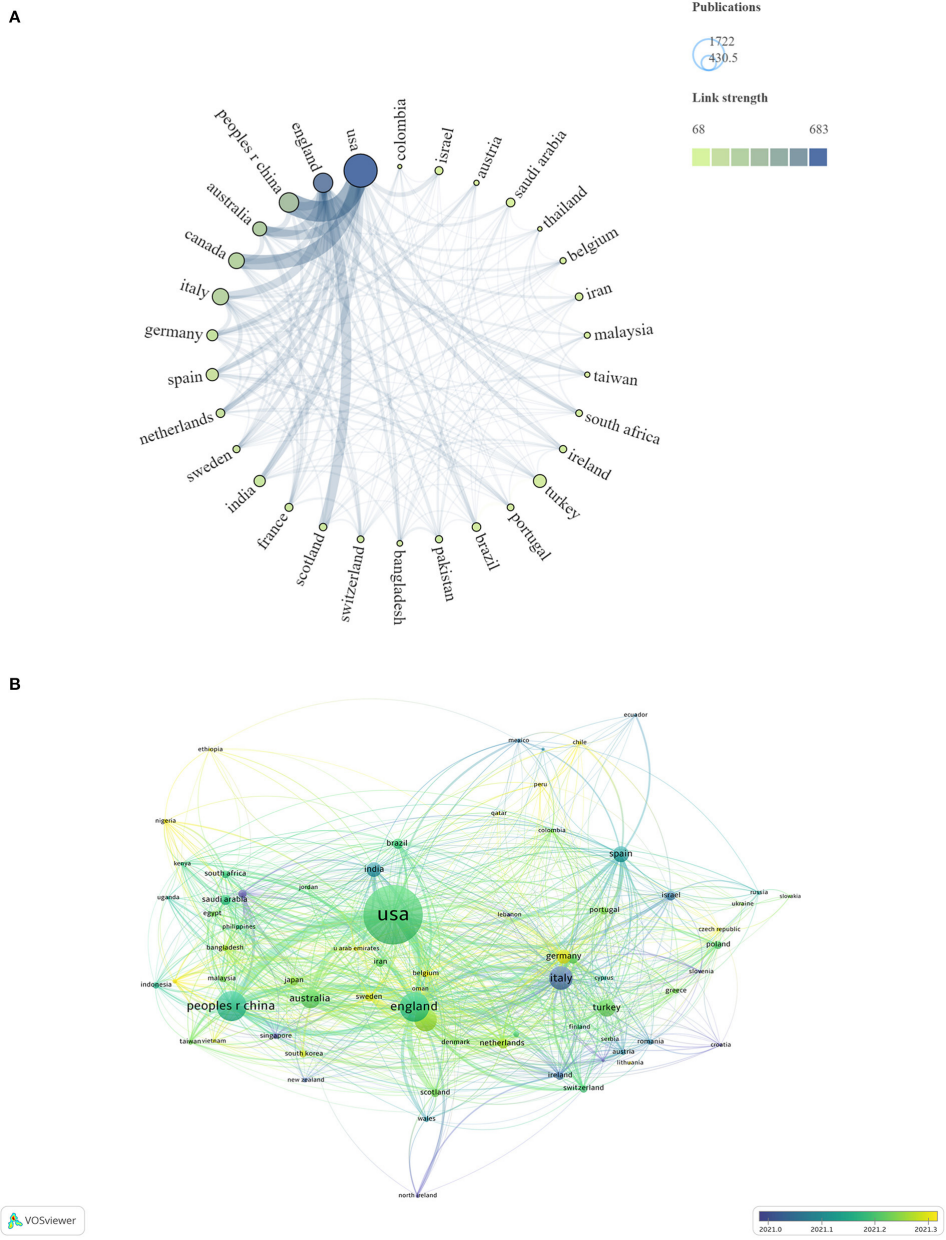
Geographical distribution in the past 3 years **(A)**. The size of the circle displays the number of publications, and the thickness of links represents cooperation strength between countries. Overlay Geographical visualization was based on publication-weights and average publication year scores **(B)**.

A total of 144 countries or regions were involved in the study of mental health of children and adolescents during COVID-19 pandemic. Data on national collaborations were exported through VOSviewer software, with a minimum number of 50 articles per country, and the national collaboration network was mapped through Scimago Graphica software as shown in Figure 2A.

Table 1 and Figure 2B shows the relationship between the number of articles published and the average time of publication in different countries. The Italian research started earlier, while the studies in China, United States, and United Kingdom were relatively later, but have a higher volume of publications and play an important role in the core collaborative network.

**Table 1:** Top 10 countries/regions according to publications.

**Rank**	**Country/regions**	**Publications (%)**	**TC**	**AC**	**Avg. pub. year**
1	USA	1,722 (35.23%)	15,800	9.18	2021.19
2	China	593 (12.13%)	9,503	16.03	2021.16
3	England	584 (11.95%)	8,610	14.74	2021.18
4	Italy	416 (8.51%)	5,243	12.60	2021.03
5	Canada	387 (7.92%)	5,839	15.09	2021.25
6	Australia	306 (6.26%)	3,049	9.96	2021.21
7	Turkey	259 (5.30%)	1,340	5.17	2021.22
8	Spain	234 (4.79%)	2,243	9.59	2021.11
9	India	197 (4.03%)	2,324	11.80	2021.09
10	Germany	190 (3.89%)	2,160	11.37	2021.29

TC, total citations; AC,average citations per paper; Avg. pub. year, the score of average publication year.

#### 3.2.2. Distribution of institutions

**Figure 3:**
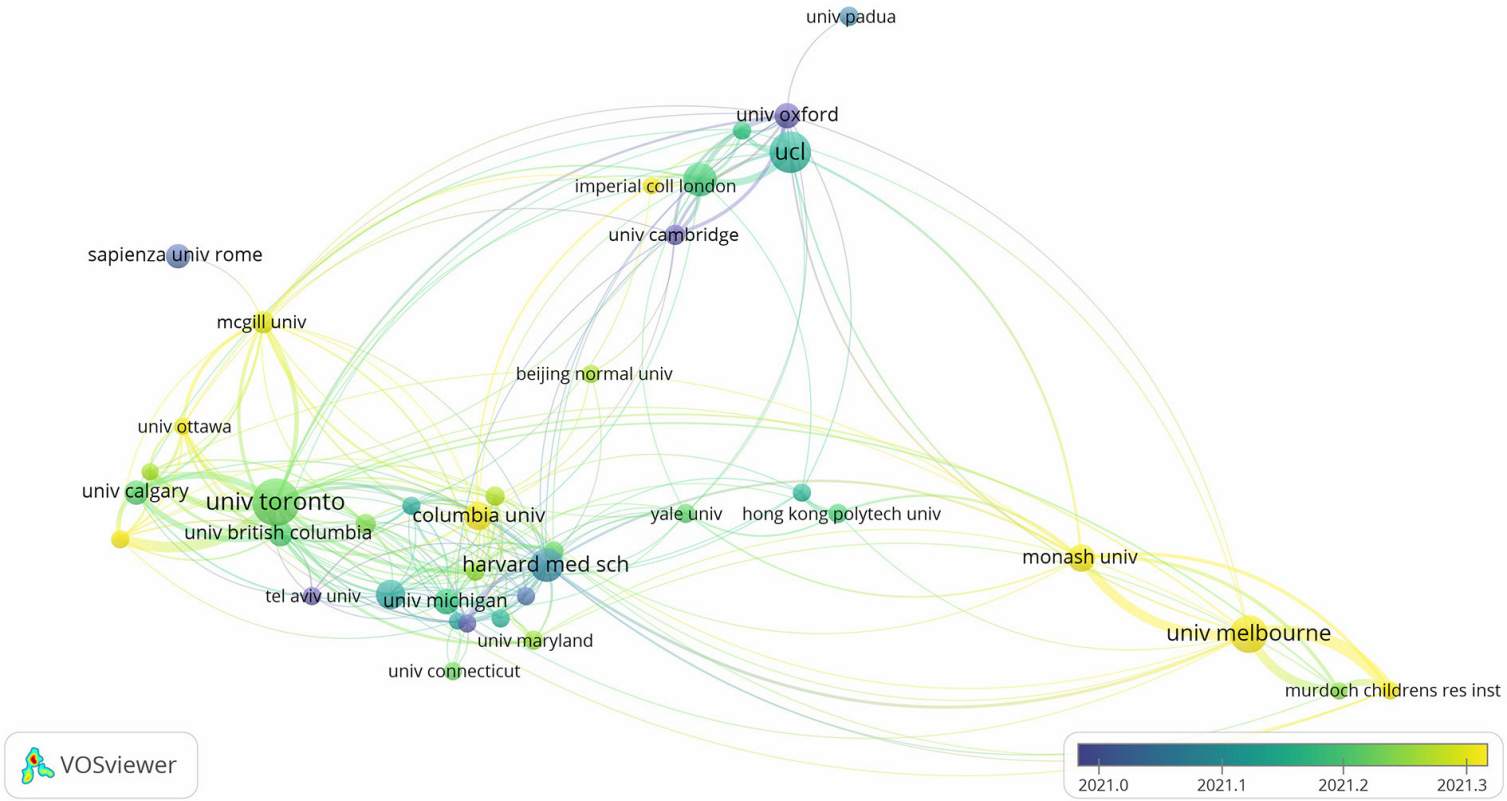
Visualization map of cooperation network between institutions, based on the publication-weights and average publication year scores.

To demonstrate the collaboration network among institutions, we used VOSviewer software for visualization mapping, and the minimum number of papers was set to 30. The relationship between the number of papers and the average publishing time of institutions is shown in Table 2 and Figure 3. A total of 39 institutions were selected, and the width of the connecting line represents the number of collaborations. The University of Toronto, University of London, and Harvard Medical School have a large number of publications and average number of citations, started their research earlier, and have closer links with other institutions in the map. Among the top ten institutions in terms of the number of publications, University of Melbourne is the latest to start its research.

**Table 2:** Top 10 productive institutions according to publications.

**Rank**	**Institution**	**Publications (%)**	**TC**	**AC**	**Avg. pub. year**
1	UNIV TORONTO	100 (15.43%)	2,959	29.59	2021.22
2	UCL	85 (13.12%)	2,102	24.73	2021.14
3	UNIV MELBOURNE	75 (11.57%)	664	8.85	2021.33
4	HARVARD MED SCH	66 (10.19%)	728	11.03	2021.07
5	KINGS COLL LONDON	65 (10.03%)	1,107	17.03	2021.19
6	UNIV WASHINGTON	56 (8.64%)	573	10.23	2021.12
7	COLUMBIA UNIV	55 (8.49%)	402	7.31	2021.31
8	MONASH UNIV	52 (8.02%)	475	9.13	2021.30
9	UNIV MICHIGAN	47 (7.25%)	413	8.79	2021.18
10	UNIV OXFORD	47 (7.25%)	566	12.04	2020.89

### 3.3. Distribution of author

**Figure 4:**
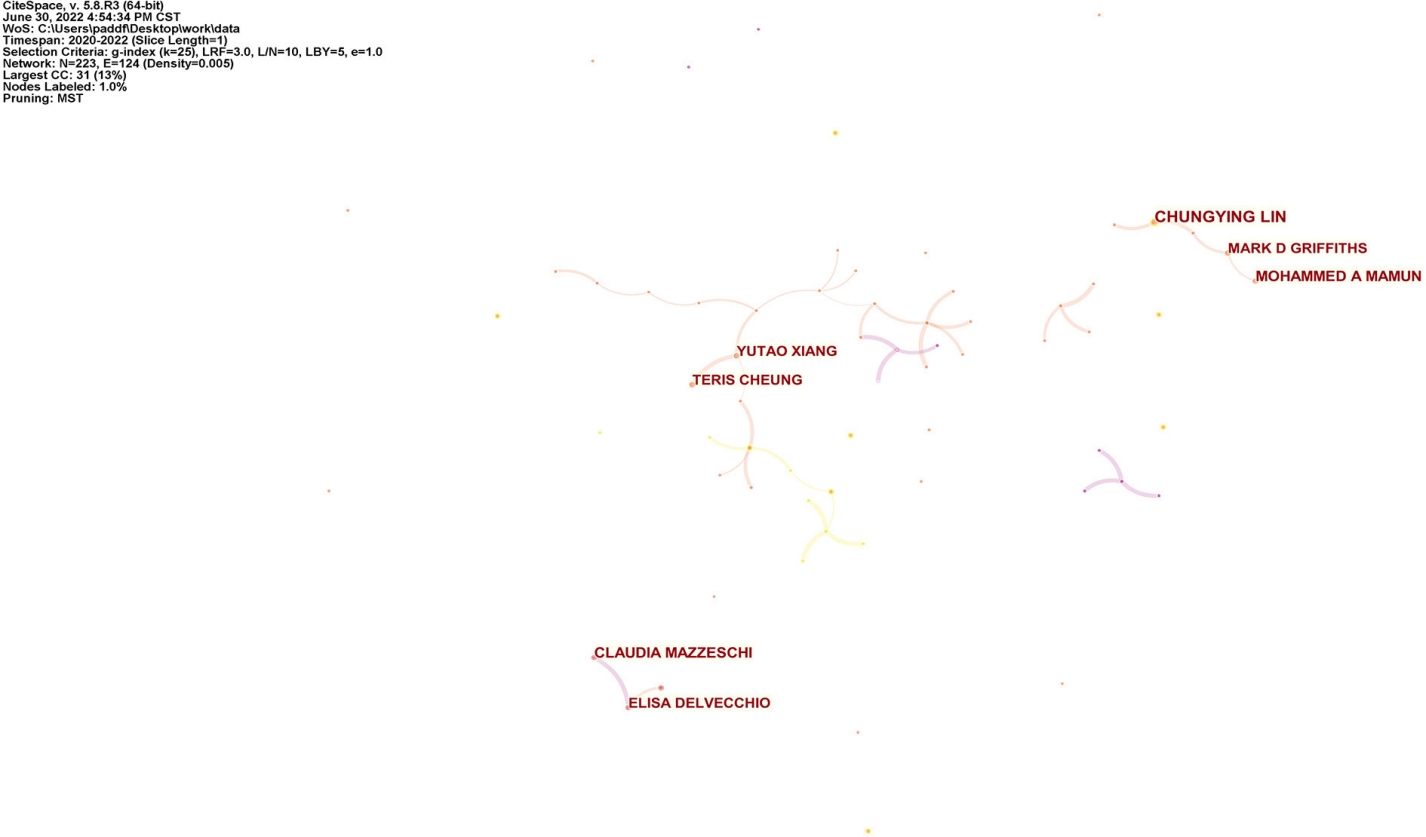
Visualization map of author network. The size of the circle displays the number of publications of this author.

We counted the publications of different authors and created a visualization map of the author collaboration network by Citespace software, as shown in Table 3 and Figure 4, with the selection criteria set as G-index of 25. G-index, as a derivative of H-index, is more sensitive than H-index in measuring the impact of researchers (16). Table 3 shows the statistical characteristics of the top 10 productive authors, including the number of publications, author's affiliation, country, and H-index, etc. Chungying Lin was the author with the highest number of publications, having 12 articles. Mohammed A Mamun and Claudia Mazzeschi ranked second with 9 articles.

**Table 3:** Top 10 productive authors.

**Rank**	**Author**	**Publications**	**Starting year**	**Institution**	**Country**	**H-index**
1	Chungying Lin	12	2021	National Cheng Kung University	China	51
2	Mohammed A. Mamun	9	2021	Jahangirnagar University	Bangladesh	34
3	Claudia Mazzeschi	9	2020	University of Perugia	Italy	26
4	Yutao Xiang	8	2021	University of Macau	China	59
5	Teris Cheung	8	2021	Hong Kong Polytechnic University	China	28
6	Mark D. Griffiths	8	2021	Nottingham Trent University	England	167
7	Elisa Delvecchio	8	2020	University of Perugia	Italy	20
8	Nicholas Chadi	7	2021	Sainte-Justine University Hospital Centre	USA	19
9	Hui Li	7	2021	Huazhong University of Science and Technology	China	11
10	Han Qi	7	2021	Capital Medical University	China	6

## 4. Co-citation analysis

### 4.1. Co-citation journals analysis

**Figure 5:**
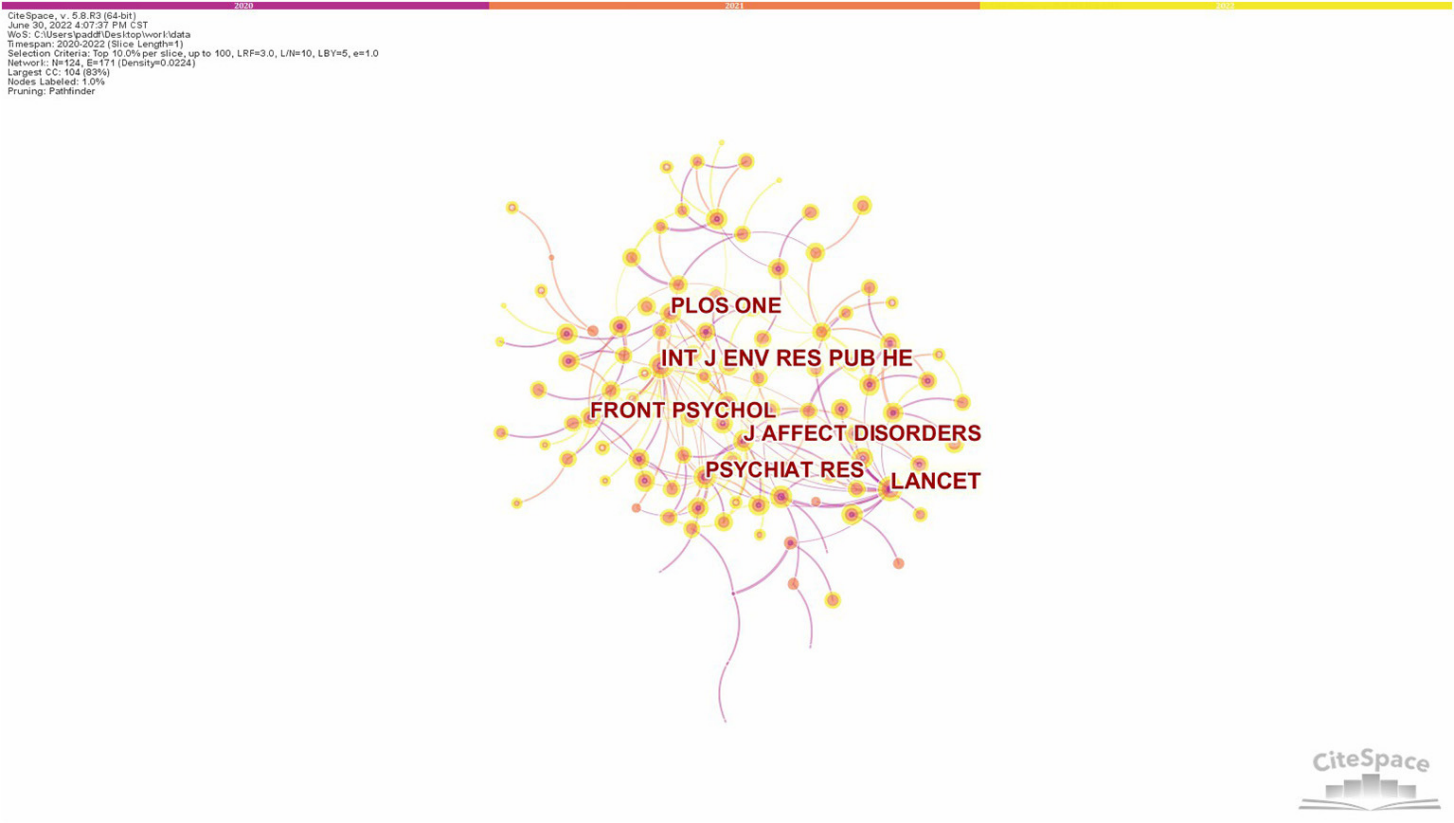
Visualization map of co-citation journals.

Co-citation is when two prior papers are cited together by another paper. Multiple co-citations can form a co-citation network, which can help to quickly understand developments in a particular field (17). Citespace software was used for co-citation analysis, and the top 10% of cited articles each year were selected for the analysis, generating 124 nodes, and the network visualization map is shown in Figure 5, where the different colored circles represent the citation volume in different years. The lines between the circles represents co-citation relationship. The thickness and number of connections between the nodes indicate the strength of links between journals. Table 4 shows the statistical parameters of the top 10 highly cited journals, including citation volume, centrality, and impact factor. Betweenness centrality is commonly used within a cluster to identify potential that could lead to transformative discoveries (11). Impact factors, although having their own limitations and drawbacks, are still an irreplaceable and quantifiable measure (18). The frequency of citations and centrality of INT J ENV RES PUB HE, hosted by Switzerland, and LANCET, hosted by the UK, are higher than other journals. The top eight journals are all cited more than 1,000 times with an average impact factor of 39.98 with high influence in this field.

**Table 4:** Top 10 highly cited journals.

**Rank**	**Journal**	**Citations**	**Centrality**	**Impact factor (2021)**	**Host country/region**
1	Int J Env Res Pub He	1,925	0.47	4.614	Switzerland
2	Lancet	1,918	0.45	202.731	England
3	Psychiat Res	1,764	0.37	11.225	Ireland
4	PLOS ONE	1,735	0.11	3.752	USA
5	Front Psychol	1,476	0.19	4.232	Switzerland
6	J Affect Disorders	1,384	0.22	6.533	Netherlands
7	Lancet Psychiat	1,116	0.11	77.056	England
8	Pediatrics	1,027	0.07	9.703	USA
9	Lancet Child Adolesc	938	0.1	37.746	England
10	Jama-J Am Med Assoc	895	0	157.335	USA

### 4.2. Co-citation documents analysis

**Figure 6:**
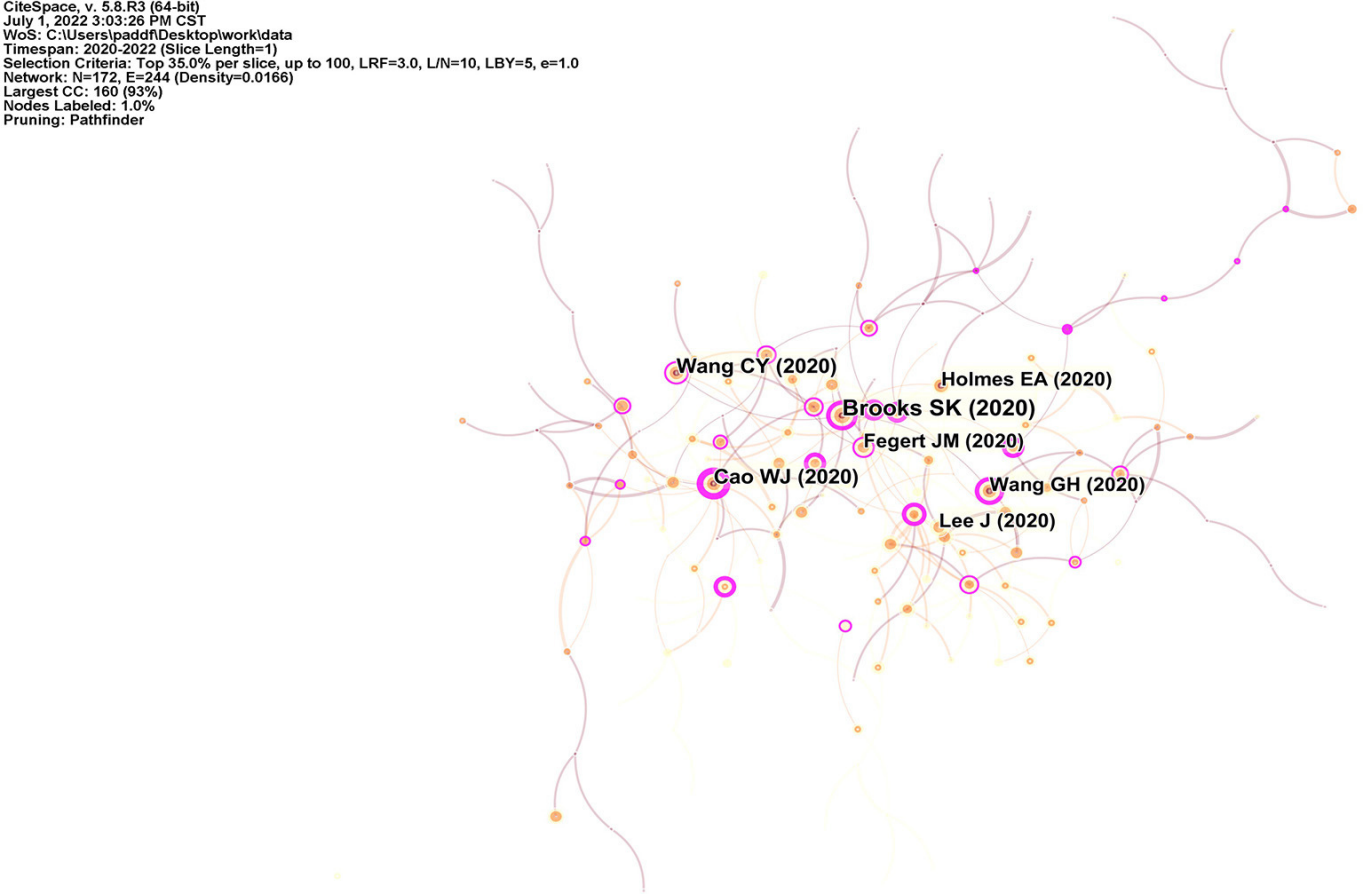
Visualization map of co-citation documents. Nodes refer to citations, and links represent co-citations between different documents.

Citespace software was used to produce the literature co-citation network shown in Figure 6. The top 35% of the publications cited per year were selected for analysis. Table 5 lists the top ten highly cited articles. *The psychological impact of quarantine and how to reduce it: rapid review of the evidence* is the most highly cited, with 680 citations. In this article, Brooks et al. evaluated measures during quarantine in 24 papers on the psychological effects of quarantine, including studies of children and adolescents (19), and proposed responses to reduce the psychological effects of quarantine, including the need for effective communication of information, the need for those in quarantine to be informed, and compliance with clear rules and regulations. *Immediate Psychological Responses and Associated Factors during the Initial Stage of the 2019 Coronavirus Disease (COVID-19) Epidemic among the General Population in China* published by Wang et al. was cited 383 times. The paper presents a retrospective cross-sectional study of students facing high levels of stress, anxiety and depression due to uncertainty about school closures (20). However, snowball sampling was used in this study, and the questionnaire was first distributed to college students, who then spread it to people around them to fill out, making the sample less representative and still in need of further study. In *The psychological impact of the COVID-19 epidemic on college students in China*, Cao et al. conducted a cross-sectional study of college students and found that economic impact, impact on daily life, and delay in academic activities were risk factors for anxiety symptoms, while stable family income and co-residence with parents were protective factors (21). The subjects of this study were university students in medical school, and since their knowledge of the novel coronavirus is more advanced than that of the general student population, whether there is a difference in anxiety status from other students still needs to be examined. In general, there were more research articles among the high cited articles, with two of the top three articles conducting cross-sectional studies; the review articles lacked systematic reviews that used critical thinking to conduct the review. Researchers are primarily concerned with the measures currently in place and the economic and social conditions in which they live.

**Table 5:** Top 10 highly cited documents.

**Rank**	**Title**	**Citation frequency**	**Centrality**	**Author**	**Type**	**Year**
1	The psychological impact of quarantine and how to reduce it: rapid review of the evidence	680	0.38	Brooks SK	Review	2020
2	Immediate Psychological Responses and Associated Factors during the Initial Stage of the 2019 Coronavirus Disease (COVID-19) Epidemic among the General Population in China	383	0.18	Wang CY	Article	2020
3	The psychological impact of the COVID-19 epidemic on college students in China	375	0.46	Cao WJ	Article	2020
4	Multidisciplinary research priorities for the COVID-19 pandemic: a call for action for mental health science	292	0.05	Holmes EA	Review	2020
5	Challenges and burden of the Coronavirus 2019 (COVID-19) pandemic for child and adolescent mental health: a narrative review to highlight clinical and research needs in the acute phase and the long return to normality	286	0.13	Fegert JM	Review	2020
6	Mitigate the effects of home confinement on children during the COVID-19 outbreak	283	0.21	Wang GH	Article	2020
7	Features Mental health effects of school closures during COVID-19	274	0.02	Lee J	Article	2020
8	Rapid Systematic Review: The Impact of Social Isolation and Loneliness on the Mental Health of Children and Adolescents in the Context of COVID-19	271	0.15	Loades ME	Review	2020
9	Behavioral and Emotional Disorders in Children during the COVID-19 Epidemic	267	0.1	Jiao WY	Article	2020
10	Prevalence and socio-demographic correlates of psychological health problems in Chinese adolescents during the outbreak of COVID-19	242	0.32	Zhou SJ	Article	2020

### 4.3. Cluster of co-citation document

**Figure 7:**
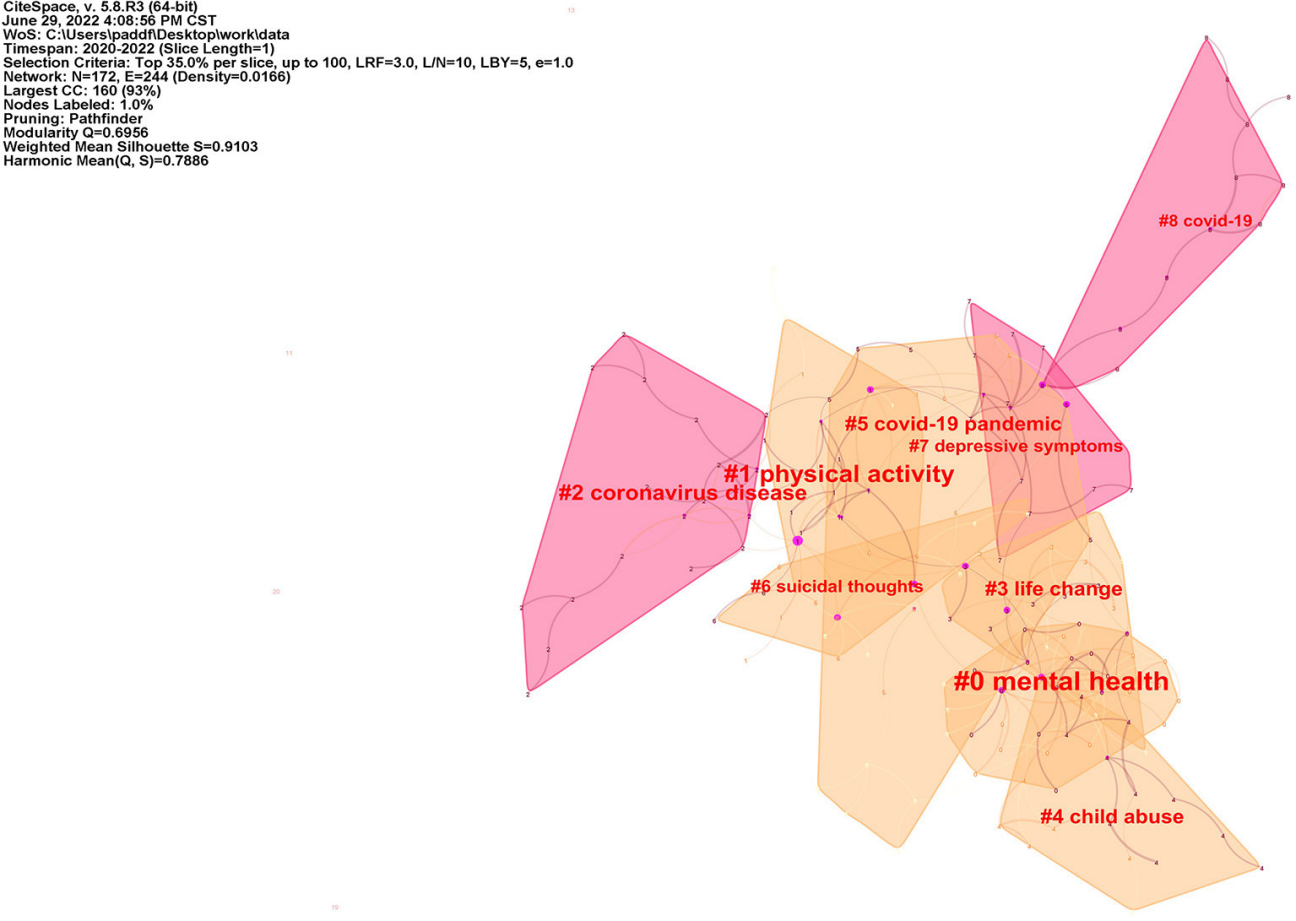
Cluster of co-citation document network.

Cluster analysis of highly cited literature was performed by Citespace software, as shown in Figure 7. The time slice was set to 1 year, and the top 35% of the literature in each slice was selected. Each cluster label was extracted from the titles of the articles cited in the cluster by Latent Semantic Indexing (LSI) method. Too much or too little cluster results can affect the clarity and accuracy of the network (9). In order to evaluate the effect of network mapping, two metrics are set, the modularity and the silhouette, and the cluster's silhouette value above 0.7 is generally convincing (22). The silhouette value of the clusters in this study is 0.9103 and the modularity value is 0.6956, which is a good fit. The larger the area of the clusters represents the more attention the researcher has paid to the field, and the intersection between clusters means that the article covers two or more fields. COVID-19 pandemic (#5) has an area of intersection with most of the remaining clusters, indicating that all studies were conducted primarily focusing on mental health issues following the COVID-19 pandemic. Mental health (#0) has a large intersection with life change (#3), child abuse (#4), indicating that researchers believe that life change and child abuse are important influential factors affecting mental health of children and adolescents (23–25). Physical activity (#1) and suicidal thoughts (#6) partially overlap, and several studies have found both decreased physical activity and increased suicidal ideation in children and adolescents during COVID-19 pandemic (26), therefore reduced outdoor time may be associated with poor mental health symptoms such as suicidal ideation (27), but the exact degree of association still needs further study. Meanwhile, some researchers investigated the suicide rate of children and adolescents during the first wave of COVID-19 pandemic on school closure in Japan and found no significant increase (28), but it remains to be studied whether the duration of quarantine and the number of repeated experiences of quarantine affect the mental health status of children and adolescents as COVID-19 is continuously prevalent in various regions. Nine representative clusters with the largest area are summarized in Table 6. Representative clusters from the nine clusters are selected for discussion below.

**Table 6:** The largest 9 clusters by size.

**Cluster ID**	**Size**	**Silhouette**	**Label (LSI)**
0	29	0.921	Mental health
1	24	0.935	Physical activity
2	19	0.929	Coronavirus disease
3	17	0.851	Life change
4	17	0.963	Child abuse
5	16	0.816	COVID-19 pandemic
6	13	0.89	Suicidal thoughts
7	13	0.874	Depressive symptoms
8	12	1	COVID-19

Class 1: mental health (#0). This cluster has the largest area with a silhouette value of 0.921 and contains 29 publications. The researchers focused on the occurrence of psychiatric symptoms in children and adolescents during COVID-19, summarized the associated risk and protective factors found, and gave relevant recommendations (5). The Delta variant of SARS-CoV-2 is spreading rapidly worldwide and the strain is extremely infectious (29), which means that more confirmed cases will emerge and people are faced with repeated quarantine for observation. a, while children and adolescents had increased reporting rates of depressive symptoms, anxiety symptoms during and after mandatory quarantine (5, 30, 31); however, a review of several studies by Racine et al. (32) found large differences in the conclusions reached by different scholars. Based on the above analysis of the highly cited literature, this phenomenon may be attributed to the poor representativeness of study sample selection, poor randomization, the suddenness of the COVID-19 outbreaks, the lack of baseline data before COVID-19 outbreak, and the short time span of the studies and the lack of studies on changes in symptoms due to the lapse of time. Therefore, more targeted, representative studies are still needed.

Class 2: life change (#1 and #3). The silhouette values were 0.935 and 0.851, respectively, and the number of highly cited articles was 24 and 17. The COVID-19 pandemic has significantly changed the lifestyle of children and adolescents, with schools closed indefinitely, online classes, parents infected with the novel coronavirus, and even children and adolescents infected or at risk of infection needing to be quarantined. The impact of this policy on their mental health is undoubtedly enormous and can even cause post-traumatic stress symptoms (19). Multiple studies have shown that COVID-19 stressors on families, including financial stressors, intimate relationship breakdowns, and psychosocial effects, may directly affect the mental health of both parents and children, with the two interacting with each other, with almost one in ten families reporting a concurrent deterioration in the mental health of children and parents (3, 21, 33–35). Severe Acute Respiratory Syndromes (SARS), a pandemic disease, was associated with an increased risk of mental illness and suicide after 12 years of follow-up of study subjects with an HR of 2.805 (36). COVID-19 has similar characteristics to its widespread prevalence, however, the extent to which these risk factors or protective factors are dangerous to the mental health of children and adolescents still needs to be specifically and systematically reviewed.

### 4.4. Timeline of keyword clusters

**Figure 8:**
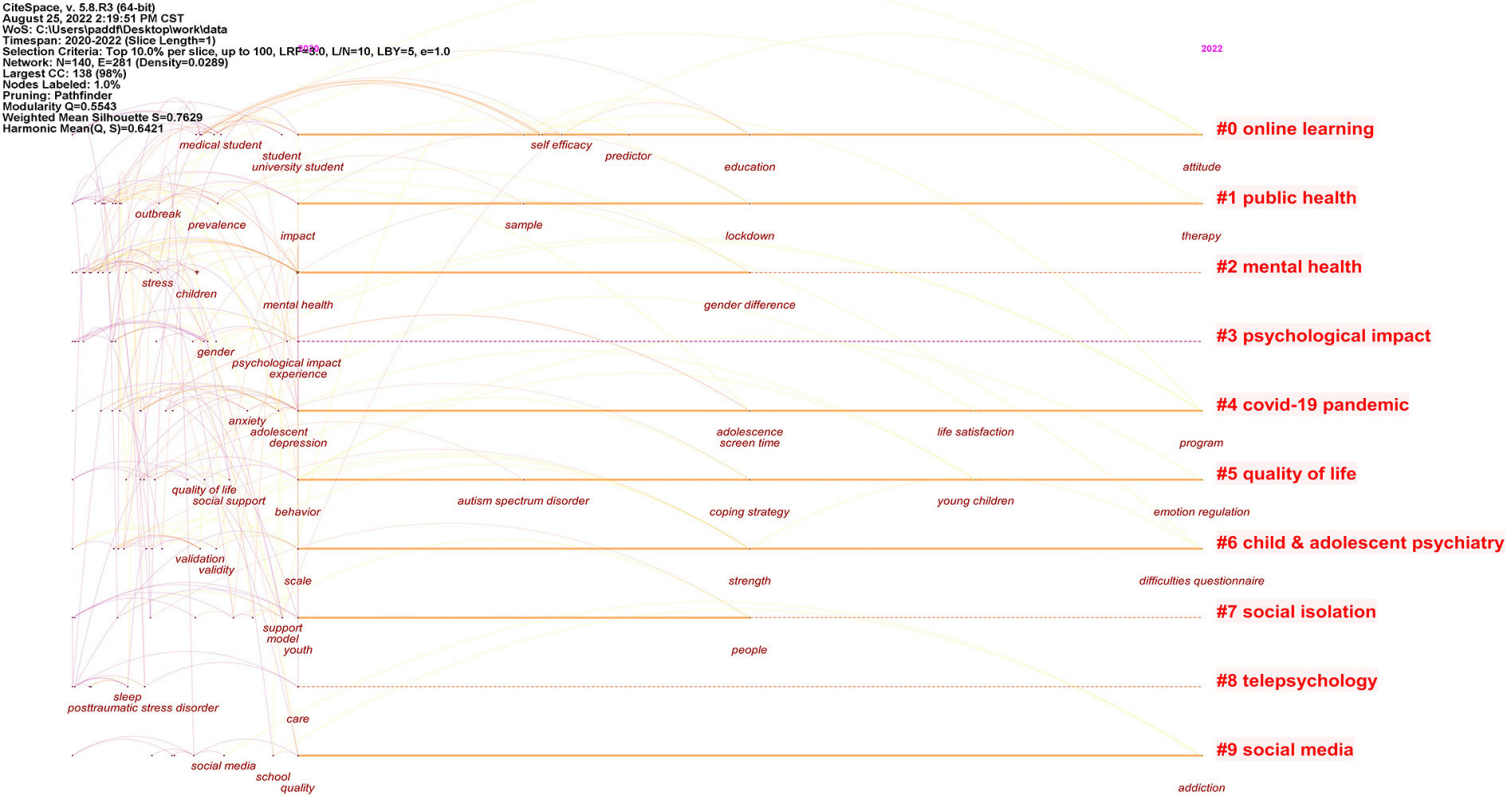
Timeline visualization of keyword clusters.

Figure 8 shows a keyword timeline analysis of the cluster network formed by the top 10% of citations per year. Table 7 lists the details of the 10 largest clusters in terms of area. Online learning (#0), Public health (#1), and Mental health (#2) were the three largest clusters in terms of area, demonstrating the highest frequency of occurrence of these three keywords in the researcher's study.

**Table 7:** The largest 10 clusters by size.

**Cluster ID**	**Size**	**Silhouette**	**Label (LSI)**	**Mean (year)**
0	17	0.69	Online learning	2020
1	17	0.6	Public health	2020
2	17	0.899	Mental health	2020
3	16	0.892	Psychological impact	2020
4	15	0.818	COVID-19 pandemic	2020
5	13	0.701	Quality of life	2020
6	12	0.758	Child and adolescent psychiatry	2020
7	11	0.728	Social isolation	2020
8	11	0.671	Telepsychology	2020
9	9	0.884	Social media	2020

Also, we summarized the objectives and keywords of the researcher's study. The drivers for the researchers focused on which social, family, or personal influences caused by the COVID-19 pandemic could have positive or negative effects on the mental health of children and adolescents, such as strict isolation measures, school closures, increased use of mobile media devices, changes in food and physical activity habits, changes in socialization patterns, domestic violence, and child abuse. Meanwhile, other authors explored what the mental health effects of COVID-19 on children and adolescents include, such as the prevalence of depression and anxiety symptoms, and made targeted recommendations.

## 5. Discussions and conclusions

Based on the literatures on mental health issues of children and adolescents during the COVID-19 pandemic, this study discusses the temporal trends and geographical distribution of the literature published on mental health issues of children and adolescents during the COVID-19 pandemic using bibliometric and visualization methods, analyzes the influence of journals, institutions, and authors in this research direction, and discusses research hotspots and development trends. We can derive the following findings.

(1) Since the outbreak of SARS-CoV-2, the research time is still short, and the research on the mental health problems of children and adolescents after the COVID-19 pandemic is still in the initial stage, but the attention to the problem is continuously increasing.
(2) From the perspective of geographical distribution, a total of 144 countries or regions were involved in the study of child and adolescent mental health during the COVID-19 pandemic, with the United States having the highest number of publications. Research on mental health issues in children and adolescents during the pandemic in the United States, China, the United Kingdom, and Italy has played an important role in the core collaborative network. Among the top ten countries in terms of number of articles published, Germany started its research later. While China has the highest average number of citations for each article which is acknowledged by a larger number of scholars.
(3) By analyzing the cooperation network among institutions, we can see that three institutions, University of Toronto, University of London, and Harvard Medical School, have a high volume of publications, while University of Melbourne, which started its research late, cooperates more closely with the above three institutions with 75 publications, ranking fourth, therefore it may make more contributions to this field in the future.
(4) By counting the publications of different authors, Chungying Lin is the first in terms of the number of publications, whose article published in 2020 focused on children's internet behavior and psychological distress during COVID-19 suspension, with a baseline before the new crown outbreak and a follow-up 5 months after the baseline, suggesting that parents should focus on problematic mobile apps and social media use (37). This author also studied mental health problems in other populations during COVID-19 and proposed several related scales. Mohammed A Mamun and Claudia Mazzeschi are ranked second in terms of their contributions. Five of the top ten authors are from China, indicating that Chinese scholars are contributing more to this field. The top ten scholars focused on mental health issues such as internet addiction due to cell phone use among children and adolescents during COVID-19 and fear due to the SARS-CoV-2 epidemic.
(5) Co-citation analysis of journals, the citation frequency and centrality of INT J ENV RES PUB HE, hosted by Switzerland, and LANCET, hosted by the UK, were higher than other journals. Three of the above ten highly cited journals were from the United States and three from the United Kingdom, the same results as the above analysis of the distribution of descriptive characteristics of countries or regions, revealing that the United States and the United Kingdom-based research groups were the main areas of concentration of child and adolescent mental health research during COVID-19 pandemic. The literature co-citation analysis concluded that there were more research articles among the highly cited articles, with two of the top three articles conducting cross-sectional studies; the review articles lacked systematic reviews that used critical thinking for review. Researchers mainly focus on the measures currently implemented and the economic and social conditions in which they are located.
(6) A cluster analysis of highly cited literature was conducted to reveal the knowledge base and research hotspots of mental health issues in children and adolescents during the COVID-19 pandemic. A total of nine clusters were obtained in this area of research, among which mental health and life change were the most representative. As the pandemic continues to spread globally, physical and psychological symptoms in children and adolescents increase over time (38), and the current review of factors that contribute to mental health and life change in children and adolescents due to the COVID-19 pandemic found that the study sample is not representative enough, whether the impact is the same for children of different genders and ages, and how large the specific impact of different factors is needs further research is needed.
(7) A cluster analysis of keywords from the top 10% of articles cited each year and a timeline view was developed to reveal keyword clusters and temporal changes during the COVID-19 pandemic targeting mental health issues in children and adolescents. A total of ten clusters were obtained. Due to the need for closure and quarantine in schools, students have had to turn to online instruction, and several studies have shown that children who are taught by remote learning are at higher risk for psychological problems and are more likely to have problems at older ages (39–42). Public health (#1) and Mental health (#2), on the other hand, can be merged into one category, with researchers' focus shifting from blocking isolation (43, 44) to what policies to put in place to maximize therapy (45–47), and with studies finding gender differences in mental health problems among children and adolescents, but different researchers have come up with opposite effects of gender on mental health (48–50), which still needs to be further explored.

While studying the physical health risks associated with COVID-19, mental health issues should not be overlooked, especially when children and adolescents are at an important stage of growth and development, requiring us to continue to conduct more in-depth studies in frontier areas and hotspot directions. Our findings are generally consistent with the results of cross-sectional studies on the impact of pandemic (51, 52), these studies reported the negative impact of the COVID-19 pandemic in children and adolescents with mental health problems. The current global pandemic of COVID-19 warns us that the emergence and spread and dissemination of pathogens are unpredictable, and our findings suggest that children and adolescents with mental health risks need appropriate social support at an earlier period to avoid mental health problems from developing into diseases. In-depth research in this direction will play an important guiding role in the future response to epidemics, disasters, and other public health emergencies in the protection of children and adolescents' mental health.

Finally, there are several limitations of this study that need to be acknowledged. The bibliometric analysis and visualization can bring effective clustering results in terms of authors, locations, and hotspot directions. However, due to the broad coverage of the subject matter selected in this paper, we enriched the selection of search terms to make the data source more accurate, nevertheless, we searched only one source, the web of science core collection, and included only English literature, thus inevitably leading to some omissions. In addition, due to the space limitation of this paper, we cannot state all the results and details of the analysis in this paper. It is undeniable that research trends will be subject to many factors. High citation rates and journal dynamics are complex processes, and the current results do not necessarily reflect true trends, which need to be further discussed in the context of multiple factors. In spite of this, this paper is not only an important reference for child and adolescent mental health researchers to better understand the current state of research on child and adolescent mental health issues in the context of the epidemic, but also a theoretical basis for further research and decision making and a technical guide for systematic reviews.

## Data availability statement

The original contributions presented in the study are included in the article/supplementary material, further inquiries can be directed to the corresponding author.

## Author contributions

ZG and YZ acquired, analyzed the data, and drafted the manuscript. ZG analyzed the data. ZG, YZ, and QL designed the research and revised the manuscript. All authors agreed to be accountable for the content of the work. All authors contributed to the article and approved the submitted version.
